# The Diversity of Meningococcal Carriage Across the African Meningitis Belt and the Impact of Vaccination With a Group A Meningococcal Conjugate Vaccine

**DOI:** 10.1093/infdis/jiv211

**Published:** 2015-04-09

**Authors:** The MenAfriCar consortium

**Affiliations:** Faculty of Infectious and Tropical Disease, London School of Hygiene and Tropical Medicine, United Kingdom

**Keywords:** Africa, meningitis, meningococcus, *Neisseria meningitidis*, carriage

## Abstract

***Background.*** Study of meningococcal carriage is essential to understanding the epidemiology of *Neisseria meningitidis* infection.

***Methods.*** Twenty cross-sectional carriage surveys were conducted in 7 countries in the African meningitis belt; 5 surveys were conducted after introduction of a new serogroup A meningococcal conjugate vaccine (MenAfriVac). Pharyngeal swab specimens were collected, and *Neisseria* species were identified by microbiological and molecular techniques.

***Results.*** A total of 1687 of 48 490 participants (3.4%; 95% confidence interval [CI], 3.2%–3.6%) carried meningococci. Carriage was more frequent in individuals aged 5–14 years, relative to those aged 15–29 years (adjusted odds ratio [OR], 1.41; 95% CI, 1.25–1.60); in males, relative to females (adjusted OR, 1.17; 95% CI, 1.10–1.24); in individuals in rural areas, relative to those in urban areas (adjusted OR, 1.44; 95% CI, 1.28–1.63); and in the dry season, relative to the rainy season (adjusted OR, 1.54; 95% CI, 1.37–1.75). Forty-eight percent of isolates had genes encoding disease-associated polysaccharide capsules; genogroup W predominated, and genogroup A was rare. Strain diversity was lower in countries in the center of the meningitis belt than in Senegal or Ethiopia. The prevalence of genogroup A fell from 0.7% to 0.02% in Chad following mass vaccination with MenAfriVac.

***Conclusions.*** The prevalence of meningococcal carriage in the African meningitis belt is lower than in industrialized countries and is very diverse and dynamic, even in the absence of vaccination.

For >100 years, large, seasonal epidemics of meningococcal meningitis have occurred periodically in the African meningitis belt [1], causing thousands of cases and many deaths and severely disrupting health systems [2]. Meningitis and septicemia are rare outcomes of meningococcal infection, and most infections with *Neisseria meningitidis* result in asymptomatic pharyngeal carriage. Therefore, the underlying transmission dynamics can be determined accurately only by study of carriage and disease, a feature of *N. meningitidis* infection first recognized 70 years ago [3].

In high-income countries, meningococcal carriage occurs most frequently in older children and young adults and is linked to smoking, nightclub attendance, and intimate kissing [4]. Less is known about the epidemiology and risks factors of meningococcal carriage in the African meningitis belt. A review of studies undertaken in the meningitis belt prior to 2007 showed that the prevalence of meningococcal carriage varied markedly between surveys, as did the distribution of serogroups [5]. Some of these differences may have arisen because surveys were undertaken at different times of the year and in different age groups and because they used a variety of swabbing and microbiological techniques. Only a few additional African carriage studies have been reported since the 2007 review, all from Burkina Faso, and these have also shown considerable heterogeneity [6–10]. In 2009, Kristiansen et al conducted large, sequential carriage studies in participants aged 1–29 years in 1 urban and 2 rural communities in Burkina Faso [9] prior to introduction of a new serogroup A meningococcal polysaccharide/tetanus toxoid conjugate vaccine (PsA-TT; MenAfriVac). The prevalence of carriage among 20 326 participants was 4.0%, with the highest prevalence among males aged 15–19 years and females aged 10–14 years.

Because carriage is central to an understanding of the epidemiology of meningococcal infection in the African meningitis belt, the African Meningococcal Carriage Consortium (MenAfriCar) was established in 2008 to study the pattern of meningococcal carriage in this region before and after the introduction of PsA-TT, using standardized field and laboratory methods [11]. Here we report the findings from 20 age-stratified, cross-sectional surveys conducted in 7 countries before and in 3 countries after the introduction of PsA-TT.

## METHODS

The field and laboratory methods used in the MenAfriCar surveys have been described in detail previously [11] and are summarized here.

### Study Sites and Carriage Surveys

Carriage studies were conducted in 1 urban and 1 rural area in 7 countries in the meningitis belt (Supplementary Figure 1) during the dry seasons of 2010 and 2011 and during the rainy season of 2012. Following pilot studies [12], 3 cross-sectional surveys were undertaken in all countries except Nigeria, where only 2 surveys could be done because of increasing political instability. Prevaccination surveys in Mali, Niger, and Chad had a target of 5000 participants, and postvaccination surveys a target of 2000 participants in Mali and Niger and 6000 in Chad. The remaining surveys had a target of 2000 participants. A representative sample frame was obtained either from an updated, ongoing demographic surveillance system (DSS) or from a census conducted specifically for the study. Multistage sampling was used. Study households were randomly selected from the DSS or census. Within selected households, individuals stratified by age group (0–4 years, 5–14 years, 15–29 years, and ≥30 years) were chosen randomly until the required sample size was reached. Following receipt of written consent, standardized household and individual questionnaires enquiring about risk factors for meningococcal infection were administered.

Based on an estimated serogroup A meningococcal carriage rate of 1% in prevaccination surveys, a sample size of 2000 was estimated to be large enough to enable meaningful comparisons to be made between countries, site (urban vs rural), season, and age group. A sample of 5000 was estimated to have 80% power to show a 50% reduction in serogroup A carriage following vaccination.

### Isolation and Characterization of Meningococci

#### Swabbing Technique

Pilot studies established that swabbing both the posterior pharynx and the tonsils or just the posterior of the pharynx were equally effective methods of isolating *N. meningitidis* [12], and the former method was used in all subsequent surveys.

#### Identification of *N. meningitidis*

Pharyngeal swabs were plated directly onto modified Thayer-Martin agar plates in the field and taken to the laboratory within 6 hours of collection. Details of the conventional bacteriological techniques used to identify and serogroup meningococci [11] are provided in the Supplementary Materials. At 6 of 7 African centers, *porA* polymerase chain reaction (PCR) was undertaken to confirm the identity of isolates of *N. meningitidis* identified by routine microbiological analysis, and if this test had positive results, an A, X, and W multiplex genogrouping PCR was performed. If the result of this first multiplex PCR was negative, singleplex Y genogrouping PCR was performed.

To confirm the identity of *Neisseria* identified by culture, samples of boiled suspensions of oxidase-positive, gram-negative bacteria were sent to the University of Oxford for molecular analysis [11, 13], as described in the Supplementary Materials. Speciation was done by sequencing the ribosomal protein L6 gene *rplF* [13], and *N. meningitidis* isolates were characterized by sequencing the 2 variable regions of the *porA* gene and of the capsule-null (*cnl)* intergenic region to identify unencapsulated bacteria. Finally, genogroups were identified by using 2 multiplex real-time PCR assays to detect genes encoding capsules corresponding to the disease-associated serogroups A, B, C, W, X, and Y (giving the respective genogroup).

### Data Management

Data were managed using the Teleform system as described previously [11]. This allows study forms to be specifically designed, scanned after completion, automatically read, and then verified before being exported to a database. Local and central databases were designed to accommodate the study forms, and a separate database module was developed to automatically populate the central database with genetic laboratory results from the *Neisseria* sequence typing database (http://PubMLST.org/Neisseria) [14, 15]. Records from laboratory reports and questionnaires were linked using the unique study number assigned to each individual. Data cleaning was performed using Stata, version 12.0 (StataCorp, College Station, Texas). Any laboratory report that could not be linked to a questionnaire that contained information on the age of the participant was excluded. These exclusions are shown in Supplementary Table 1; only 18 meningococci, 15 of which were from the first survey, were excluded.

### Statistical Methods

Analyses were performed using Stata, version 12.0. We accounted for potential household clustering, using the survey commands in Stata. Overall and age-stratified carriage prevalence rates were calculated, together with 95% confidence intervals (CIs), for each country and each survey. The Simpson diversity index was calculated for each country, based on the strains (genogroup and PorA subtype) isolated across all surveys. Risk factors for carriage were assessed using logistic regression. Each risk factor was considered in turn, using univariable logistic regression. A multivariable model was then constructed that included country, season, age group, and sex a priori and any variable with a *P* value of <.1 in the univariable analyses, with those with a *P* value of <.05 in the multivariable analysis retained. As a final check, dropped variables were reentered into the model one at a time, and the *P* values were reexamined; the variable was retained if *P* < .05. A multinomial logistic regression model was constructed to compare risk factors for carriage of meningococci with and those without capsular genes.

## RESULTS

### Participants Surveyed

The number of participants in each survey, the year and season of the survey, and the relationship of the survey to the introduction of PsA-TT are shown in Table 1. Most surveys recruited close to their target number of participants, and there was even sampling between age groups (Supplementary Table 2). Participants aged 5–29 years were recruited preferentially for the postvaccination survey in Chad in 2012 to maximize the chance of identifying serogroup A meningococcal carriers and of being able to assess the impact of vaccination.

**Table 1.** Numbers of Subjects Studied, by Country, During Each Survey

Country	Survey 1, Rainy Season, July–December 2010	Survey 2, Rainy Season, July–November 2011	Survey 3, Dry Season, February–July 2012
Chad	1986	5307	6103^a^
Ethiopia	1884	2025	2061
Ghana	1159	2031	2019
Mali	4844	1994^a^	1999^a^
Niger	4235	1963^a^	2015^a^
Nigeria	1520	936	0
Senegal	1414	1680	1315
Overall	17 042	15 936	15 512

^a^ Data are from a postvaccination survey.

### Prevalence of *N. meningitidis* Carriage

**Figure 1.**
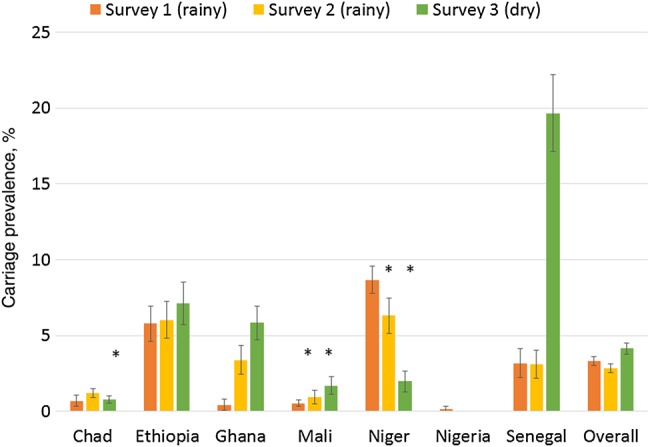
Carriage prevalence, by country and survey. Asterisks denote postvaccination surveys.

**Figure 2.**
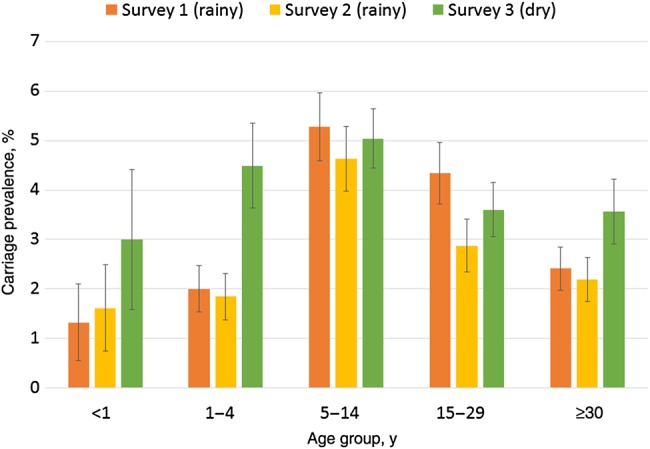
Carriage prevalence, by age and survey.

**Figure 3.**
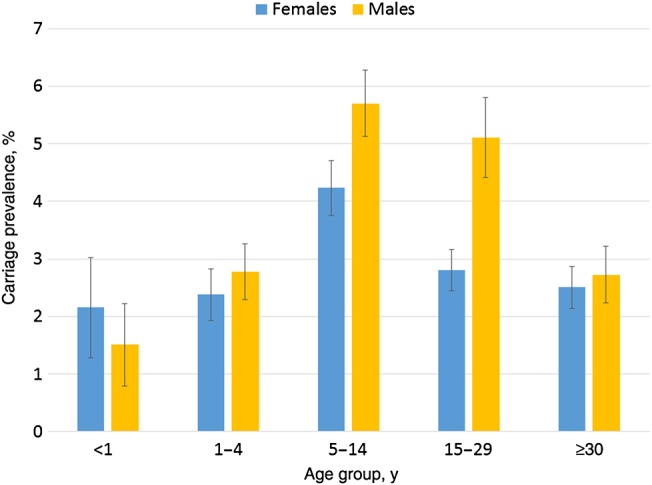
Carriage prevalence, by age and sex (all surveys combined).

A total of 1687 meningococci were identified, for an overall carriage prevalence of 3.4% (95% CI, 3.2%–3.6%) after adjustment for survey design. There was marked variation in the prevalence of carriage between centers and between surveys within each center (Figure 1). The age distribution of carriers by center and survey was more consistent (Figure 2). Combining the results from all surveys, the prevalence of carriage by age was 1.8% (95% CI, 1.3%–2.4%) for <1 year, 2.6% (95% CI, 2.2%–2.9%) for 1–4 years, 4.9% (95% CI, 4.5%–5.3%) for 5–14 years, 3.6% (95% CI, 3.2%–3.9%) for 15–29 years, and 2.6% (95% CI, 2.3%–2.9%) for ≥30 years. The age distribution of carriers by country and survey is shown in Supplementary Figure 2. The overall prevalence of carriage in males (4.1%; 95% CI, 3.8%–4.3%) was higher than in females (3.0%; 95% CI, 2.7%–3.2%), and this difference was seen in 5 of 7 participating countries. The most marked sex difference in prevalence was seen in 15–29 year olds, in whom carriage in males was significantly higher than in females (5.1% vs 2.8%; *P* < .0001; Figure 3). Generally, the prevalence of carriage was higher in the dry season (4.2%; 95% CI, 3.8%–4.5%) than in the rainy season (3.1%; 95% CI, 2.9%–3.3%); this difference was observed in 4 of 7 participating countries. The prevalence of carriage was higher in rural areas (3.6%; 95% CI, 3.3%–3.9%) than in urban areas (3.2%; 95% CI, 2.9%–3.5%), and this difference, although not statistically significant, was observed in 5 of 7 participating countries (Supplementary Table 3). The most frequently identified *Neisseria* species other than *N. meningitidis* was *Neisseria lactamica* (2588 individuals [5.4%]); other *Neisseria* species were found at a lower prevalence.

### Strain Characteristics of *N. meningitidis* Carriage Isolates

**Figure 4.**
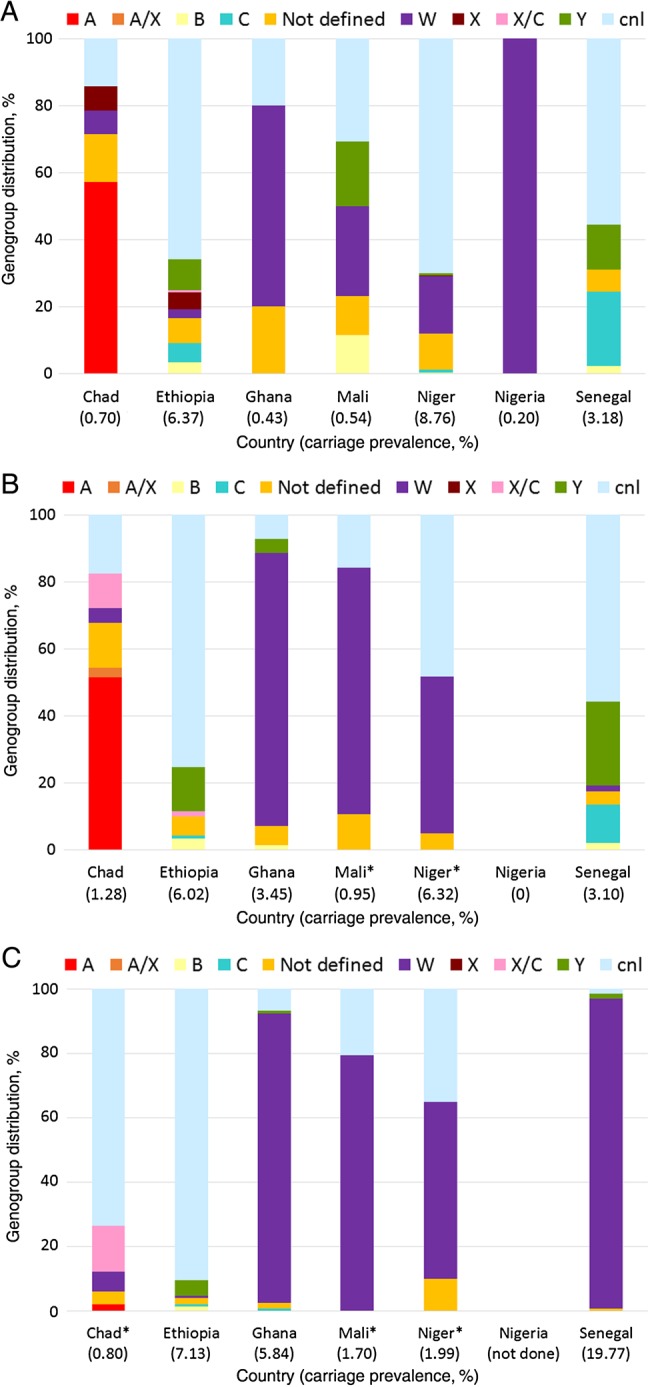
Distribution of *Neisseria meningitidis* genogroups, by country and survey. The overall prevalence of carriage in each country during the survey is shown in brackets. *A*, First cross-sectional survey in 2010 (rainy season). At this time, no country had introduced serogroup A meningococcal conjugate vaccine (MenAfriVac). *B*, Second cross-sectional survey in 2011 (rainy season). At this time, MenAfriVac had been introduced in Mali and Niger, as indicated by asterisks. *C*, Third cross-sectional survey in 2012 (dry season). At this time, MenAfriVac had been introduced in Mali, Niger, and Chad, as indicated by asterisks.

Genes characteristic of disease-associated capsules were detected in 48% of the 1687 meningococci identified: genogroup A, 46 isolates (0.09% overall prevalence); genogroup B, 17 isolates (0.04%); genogroup C, 30 isolates (0.06%); genogroup W, 623 isolates (1.3%); genogroup X, 27 isolates (0.06%); and genogroup Y, 68 isolates (0.14%). Two genogroups were identified in 3 samples: 2 with genogroup A and X, and 1 with genogroup X and C. The overall prevalence of carriage with *cnl* meningococci was 1.6% (778 isolates). Neither a capsular gene nor the *cnl* intergenic region was detected in 101 samples identified microbiologically as *N. meningitidis.* It is likely that these were meningococci with capsules not usually associated with disease and whose capsular genes were not detected [16]. In 169 samples identified as meningococci, we found evidence of both a capsule-null locus and a capsular gene. This suggests that there was cocolonization with >1 meningococcal strain or perhaps cocolonization with 2 different *Neisseria* species, as the *cnl* intergenic region is present in all nonmeningococcal *Neisseria* species, as well as some meningococci. The distribution of the different genogroups varied substantially by center and survey (Figure 4*A*–*C*). Although numbers within subgroups were small, there was no indication that that genogroup was associated preferentially with a particular age group or sex within the context of a country or survey (Supplementary Table 4).

A wide diversity of porA subtypes was recorded, with 63 unique combinations identified among the samples typed for both VR1 and VR2. However, 3 subtypes accounted for 68.8% of all the meningococci isolated. The most frequently detected subtype was P1.18-11,42-1 (n = 547), which was usually associated with the presence of the *cnl* intergenic region (93%). The next most common subtype was P1.5,2 (n = 493), which was strongly associated with genogroup W (97%), followed by P1.5-1,2–36 (n = 121), which was also associated strongly with genogroup W (89%). Forty-one of the 46 genogroup A meningococci were subtyped as P1.20,9.

**Figure 5.**
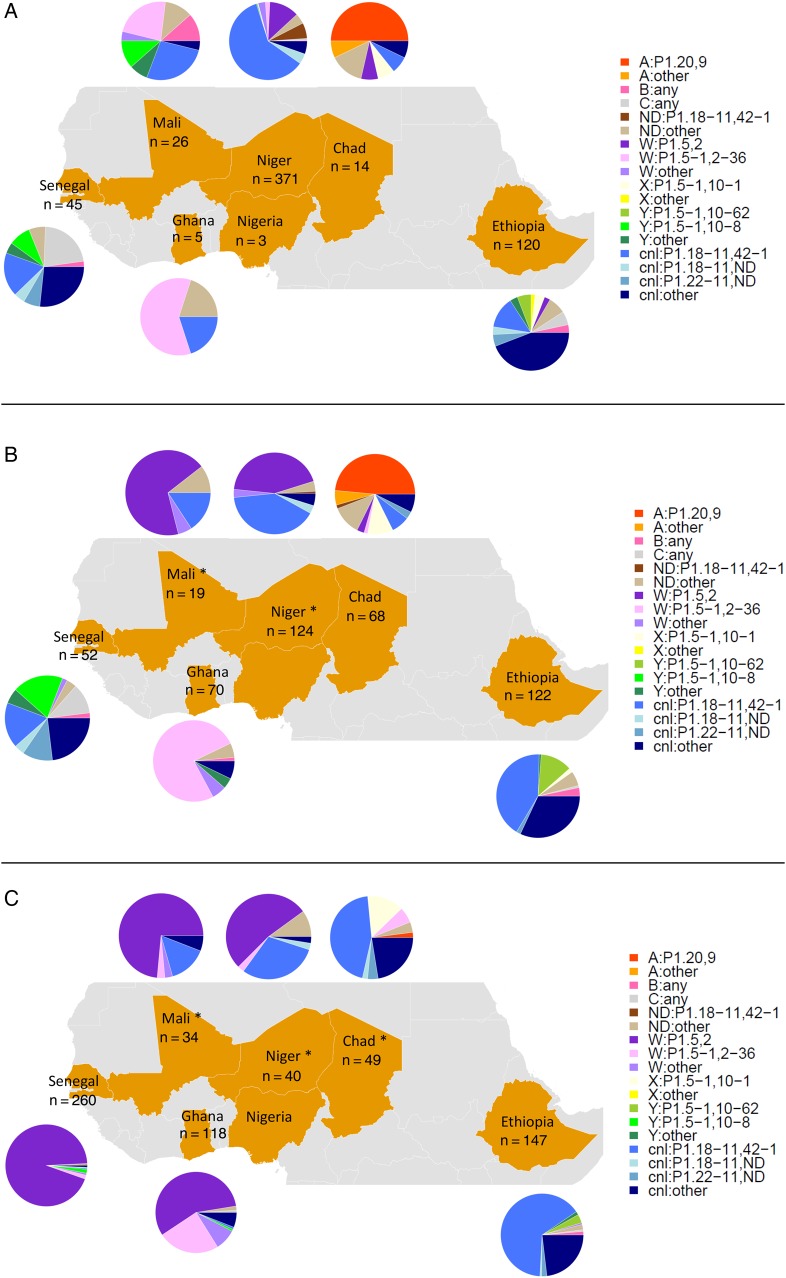
Distribution of strain types across the meningitis belt. The number of meningococci isolated in each country is indicated. *A*, Survey 1 (2010). At this time, no country had introduced serogroup A meningococcal conjugate vaccine (MenAfriVac). *B*, Survey 2 (2011). At this time, MenAfriVac had been introduced in Mali and Niger, as indicated by asterisks. *C*, Survey 3 (2012). At this time, MenAfriVac had been introduced in Mali, Niger, and Chad, as indicated by asterisks.

The combination of genogroup and PorA subtype provides a strain designation; 132 distinct strains were identified. The most frequently identified strain type was NG(*cnl*):P1.18-11,42-1 (n = 511), followed by W:P1.5,2 (n = 479) and W:P1.5-1,2-36 (n = 108). All other strain types were isolated on <50 occasions. The strain distribution by country and by survey is illustrated in Figure 5. More strain diversity was observed in Ethiopia and Senegal, countries at the eastern and western edges of the meningitis belt, than in countries situated in the central part of the belt. The Simpson diversity index (from most to least diverse) was estimated to be 0.021 in Ethiopia, 0.031 in Senegal, 0.035 in Niger, 0.051 in Ghana, 0.052 in Chad, and 0.073 in Mali. Meningococci isolated in Ethiopia were >3 times as diverse as those from Mali.

### Risk Factors for *N. meningitidis* Carriage

Risk factors for carriage with *N. meningitidis* at the individual level are shown in Table 2. Age, sex, season, rural site, crowding (≥2 people per room), smoking in the household, and indoor kitchen facilities remained statistically significant risk factors in a multivariable logistic regression analysis. Recent immunization (within 1 year) with a meningitis vaccine (self-reported) was associated with a lower odds of carriage of capsulated meningococci, after accounting for other factors. Respiratory symptoms and a history of attending social gatherings in the past week were associated with a lower odds of carriage in univariable analysis, but both variables dropped out in multivariable analysis. Risk factors for carriage of genogroupable and capsule-null meningococci are shown in Supplementary Table 5. Dry season and the location of the kitchen were associated significantly with a higher odds of carriage of genogroupable but not *cnl* meningococci.

**Table 2.** Univariable and Multivariable Logistic Regression Analysis of Factors Associated With *Neisseria meningitidis* Carriage

Factor	Subjects, No. (Carriers, No.)	Crude OR (95% CI)	Adjusted OR (95% CI)
Age, y			
<1	2199 (41)	0.50 (.37–.69)	0.37 (.27–.51)
1–4	8839 (228)	0.72 (.61–.84)	0.59 (.50–.69)
5–14	13 121 (655)	1.41 (1.35–1.58)	1.41 (1.25–1.60)
15–29	12 426 (450)	1.0	1.0
≥30	11 905 (313)	0.72 (.62–.83)	0.60 (.51–.69)
Sex^a^			
Female	27 981 (835)	1.0	1.0
Male	20 353 (825)	1.37 (1.25–1.51)	1.17 (1.10–1.24)
Season			
Rainy (XS1 and XS2)	32 978 (1039)	1.0	1.0
Dry (XS3)	15 512 (648)	1.31 (1.21–1.53)	1.54 (1.37–1.75)
Country			
Chad	13 396 (131)	1.0	1.0
Ethiopia	5970 (389)	7.12 (5.71–8.89)	7.04 (5.39–9.21)
Ghana	5209 (193)	4.03 (3.15–5.15)	4.76 (3.62–6.29)
Mali	8837 (79)	0.95 (.71–1.27)	1.42 (1.03–195)
Niger	8213 (535)	7.27 (5.93–8.93)	10.16 (8.03–12.84)
Nigeria	2456 (3)	0.09 (.02–.35)	0.11 (.03–.45)
Senegal	4409 (357)	9.23 (7.40–11.50)	9.58(7.30–12.58)
Area			
Urban	20 218 (644)	1.0	1.0
Rural	28 205 (1017)	1.14 (1.01–1.28)	1.44 (1.28–1.63)
Crowding^b^			
<2 people per room	16 908 (460)	1.0	1.0
≥2 people per room	31 515 (1201)	1.42 (1.25–1.61)	1.24 (1.09–1.42)
Smoking status^c^			
Nonsmoker	22 312 (705)	1.0	…
Smoker	1231 (43)	1.11 (.81–1.51)	…
Smokers in household			
No	35 523 (1164)	1.0	1.0
Yes	12 881 (497)	1.18 (1.04–1.34)	1.16 (1.02–1.33)
Cooking fuel			
Oil based	533 (7)	0.46 (.22–.97)	…
Wood based	41 825 (1172	1.0	…
Cow dung/straw	5729 (472)	3.11 (2.73–3.55)	…
Kitchen location			
Open air	18 934 (512)	1.0	1.0
Inside house	13 322 (824)	2.37 (2.09–2.70)	1.35 (1.12–1.63)
Separate hut	15 268 (304)	0.72 (.61–.84)	0.95 (.77–1.18)
Missing information	595 (21)	1.32 (.73–2.38)	1.11 (.60–2.07)
Vaccinated recently with meningitis vaccine			
No	33 598 (1230)	1.0	1.0
Yes, <1 y ago	9132 (227)	0.67 (.57–.78)	0.72 (.60–.85)
Yes, 1–3 y ago	4589 (171)	1.01 (.85–1.21)	0.52 (.42–.63)
Don't know/ missing	1171 (59)	0.81 (.56–1.16)	1.09 (.50–1.04)
Respiratory symptoms			
No	26 555 (1077)	1.0	…
Yes	21 935 (610)	0.69 (.62–.77)	…
Social gatherings			
None attended	20 370 (872)	1.0	…
1–2 types of gathering attended	24 719 (702)	0.65 (.59–.72)	…
≥3 types of gathering attended	3333 (87)	0.60 (.48–.75)	…

The final multivariable logistic regression model included age group, sex, country, survey, area, crowding, household smokers, recent vaccination and kitchen location.

Abbreviations: CI, confidence interval; OR, odds ratio.

^a^ Data were not reported for 135 individuals.

^b^ Data were missing for 67 individuals.

^c^ Only adults were asked about their smoking habits.

### Impact of Vaccination

Surveys were conducted before and after vaccination with PsA-TT in 3 countries (Chad, Mali, and Niger). In Chad, where an epidemic of serogroup A meningococcal disease was occurring at the time of vaccination, a marked drop in the prevalence of genogroup A meningococcal carriage was observed 4–6 months following vaccination (from 0.7% to 0.02%; *P* < .0001), as reported previously [17]. In Chad, PorA characterization of meningococcal isolates before and after vaccination showed a marked change in pattern, with a substantial reduction in the prevalence of the PorA subtype P1.20.9 associated with genogroup A (*P* < .0001), and a marked increase in both the proportion and overall prevalence of the *cnl*-associated subtype P1.18,42-1 (Figure 5). However, similar rapid changes in strain prevalence over time occurred in other countries where vaccination was not introduced during the period of the surveys, including Ethiopia and Senegal. In Mali and Niger, no genogroup A meningococcal carriers were identified during the survey conducted 4–6 months before vaccination with PsA-TT, so the impact of vaccination on genogroup A carriage could not be assessed.

## DISCUSSION

Results from 20 standardized surveys conducted by the MenAfriCar consortium in 7 countries across the African meningitis belt at approximately the same time confirm the findings of previous studies undertaken at single sites, at different times of the year, and with different techniques that meningococcal carriage in the African meningitis belt is both complex and highly dynamic, both in terms of the prevalence of carriage and meningococcal genogroup [5, 18]. This is illustrated well by the findings from Senegal, where carriage prevalence increased from around 3% in the first and second surveys to 19.8% in the third survey with the emergence of a strain type not observed in the previous surveys.

The overall prevalence of meningococcal carriage (3.4%) detected during the 20 surveys was substantially lower than that recorded in most population surveys undertaken in high-income countries with a temperate climate, where carriage prevalences 2 or 3 times greater are usually recorded [19]. Similar findings to ours were obtained recently in Burkina Faso, where surveys conducted before and after the introduction of PsA-TT showed the overall meningococcal carriage rate to be low (4% in prevaccine studies and 7% in postvaccine studies) [14]. In addition to the low prevalence of carriage found during the MenAfriCar surveys, the diversity of strain types was more restricted in the central part of the meningitis belt than would be expected in high-income countries with a temperate climate. The distribution of strain types in Butajira, Ethiopia, which does not have a climate typical of the Sahel, was more diverse and closer to that found in countries with a temperate climate than in countries at the center of the meningitis belt. Detection of a low prevalence of carriage and low strain diversity in countries of the meningitis belt seems, at first, to be counterintuitive but is consistent with the general theory that infectious diseases epidemics occur in situations where an infection is uncommon and population susceptibility is high.

The proportion of nongroupable, capsule-null *N. meningitidis* found in nearly all surveys was higher than that found in comparable studies conducted in high-income countries with a temperate climate [20, 21]. The reason and significance of this phenomenon is uncertain, but it warrants further investigation because the presence of these strains could influence infections with capsulated meningococci.

The prevalence of meningococcal carriage by age was consistent with most previous surveys conducted in Africa, with the highest value detected in individuals aged 5–14 years [5]. This age pattern is compatible with substantial transmission occurring outside households between school-aged children. Risk factors for carriage were similar to those reported previously in Africa [22] but different from those seen in other settings where carriage peaks in young adults [19]. Carriage was more prevalent in males than females, perhaps because of more-frequent social mixing among young adult males than females in Muslim societies; in rural areas than urban areas; and in those exposed to smoke, either from cigarettes or from cooking. Development of less polluting methods of cooking is a priority for many parts of the developing world [23] and could contribute to a reduction in the incidence of meningococcal infection, as well as the incidence of pneumonia. The absence of an association between carriage and recent respiratory symptoms contrasts with findings from studies in Burkina Faso [6]. The lack of any association with attendance at social gatherings was inconsistent with findings from the United Kingdom, where social behavior was closely linked to the risk of carriage [4]. However, in the present study, information on the number and types of gatherings attended but not the frequency of attendance was collected. Previous vaccination (within 3 years) was associated with a lower risk of carriage of capsulated meningococci. Since this finding relates mainly to the use of polysaccharide vaccines in Ghana (A+C polysaccharide vaccine) and Senegal (A+C+W polysaccharide vaccine) rather than the use of MenAfriVac, and because polysaccharide vaccines have little effect on carriage [24], this association is most likely due to unmeasured confounding. The odds of carriage with meningococci that possessed a capsular gene was higher in the dry season than in the rainy season, whereas there was no significant difference in the odds of carriage for unencapsulated (*cnl*) strains by season. An indoor kitchen was an additional risk factor for genogroupable meningococci, compared with capsule-null strains. These findings are consistent with the idea that the capsule protects meningococci against desiccation during aerosol transmission [25].

The MenAfriCar surveys were timed to permit the impact of the PsA-TT vaccine to be evaluated in Chad, Mali, and Niger. However, in Mali and Niger, carriers of group A meningococci were not found in prevaccination surveys, so it was not possible to measure the impact of vaccination on group A carriage in these countries. It seems unlikely that PsA-TT would have had any impact on other serogroups in Mali or Niger (serogroup replacement), because there were no serogroup A meningococci present prior to vaccination to displace. In Niger, where the recent epidemiology of meningococcal disease has been studied in detail [26], the decline in group A infections appears to have been part of a natural cycle, rather than due to vaccination. In Chad, where an epidemic of group A meningococcal disease was in progress at the time that PsA-TT was introduced in part of the country, vaccination was associated with a dramatic reduction in the prevalence of carriage of genogroup A meningococci in both vaccinated and unvaccinated participants, as reported previously [17] and as observed also in Burkina Faso [14]. However, because only data before and after vaccine introduction were obtained in both studies, and because rapid changes in carriage may occur independently of vaccination, these findings need to be considered with caution. Assessment of carriage dynamics beyond 1 year is needed to evaluate the long-term impact of PsA-TT in the meningitis belt.

An important part of the MenAfriCar project was capacity building through strengthening the ability of our study centers to undertake meningococcal carriage surveys and disease surveillance. Standardization of methods was undertaken through workshops and site visits. The Institut Pasteur (Paris, France) conducted quality-control studies for the consortium. Nevertheless, it is still possible that some of the variability in prevalence of carriage seen between centers and surveys was due to technical differences rather than to biological factors, but use of confirmatory molecular characterization of DNA samples has reduced this risk.

It is likely that widespread deployment of PsA-TT, now given to >217 million people in countries of the meningitis belt, will prevent future epidemics of serogroup A meningococcal meningitis, at least for a number of years. However, meningococci of other serogroups (C, W, and X) have the potential to cause epidemics [27], and it is uncertain what impact elimination of serogroup A meningococci will have on the frequency of outbreaks caused by these serogroups. Whether or not mass vaccination drives serogroup replacement is likely to be determined in part by the complex interactions within the bacterial ecology of the pharynx, a subject that deserves further study.

## MEMBERS OF THE STUDY GROUP

Institutions and individual members of the MenAfriCar consortium who contributed to this study are as follows: Armauer Hansen Research Institute, Addis Ababa, Ethiopia: Oumer Ali, Abraham Aseffa (principal investigator [PI]), Ahmed Bedru, Tsehaynesh Lema, Tesfaye Moti, Yenenesh Tekletsion, Alemayehu Worku, Haimanot Guebre Xabher (deceased), and Lawrence Yamuah; Centre de Recherche Médicale et Sanitaire, Niamey, Niger (member of the International Network of Pasteur Institutes): Rahamatou Moustapha Boukary, Jean-Marc Collard (PI), Ibrahim Dan Dano, Ibrahim Habiboulaye, Bassira Issaka, Jean-François Jusot, Sani Ousmane, and Issoufa Rabe; Centre de Support en Santé International, N'Djamena, Chad: Doumagoum Moto Daugla (PI), Jean Pierre Gami, Kadidja Gamougam, Lodoum Mbainadji, Nathan Naibei, Maxime Narbé, and Jacques Toralta; Centre pour les Vaccins en Développement, Bamako, Mali: Abdoulaye Berthe, Kanny Diallo, Mahamadou Keita, Uma Onwuchekwa, Samba O. Sow (PI), Boubou Tamboura, Awa Traore, and Alou Toure; Centers for Disease Control and Prevention, Atlanta, Georgia: Tom Clark and Leonard Mayer; Department of Community Medicine, University of Maiduguri, Nigeria: Mary Amodu, Omeiza Beida, Galadima Gadzama, Babatunji Omotara (PI), Zailani Sambo, and Shuaibu Yahya; Faculty of Infectious Disease, London School of Hygiene and Tropical Medicine, United Kingdom: Daniel Chandramohan, Brian M. Greenwood (PI), Musa Hassan-King, Olivier Manigart, Maria Nascimento, James M. Stuart, and Arouna Woukeu; Princeton University, New Jersey: Nicole E. Basta; Public Health England Vaccine Evaluation Unit, Manchester, United Kingdom: Xilian Bai, Ray Borrow, and Helen Findlow; Institut de Recherche pour le Développement, Dakar, Senegal: Serge Alavo, Hubert Bassene, Aldiouma Diallo (PI), Marietou Dieng, Souleymane Doucouré, Jules François Gomis, Assane Ndiaye, Cheikh Sokhna, and Jean François Trape; Navrongo Health Research Centre, Ghana: Bugri Akalifa, Abudulai Forgor, Abraham Hodgson (PI), Isaac Osei, Stephen L Quaye, John Williams, and Peter Wontuo; University of Bristol, United Kingdom: Thomas Irving; University of Cambridge, United Kingdom: Caroline L. Trotter (formerly of the University of Bristol); and University of Oxford, United Kingdom: Julia Bennett, Dorothea Hill, Odile Harrison, Martin C. Maiden, Lisa Rebbetts, and Eleanor Watkins.

## Supplementary Data

Supplementary materials are available at *The Journal of Infectious Diseases* online (http://jid.oxfordjournals.org). Supplementary materials consist of data provided by the author that are published to benefit the reader. The posted materials are not copyedited. The contents of all supplementary data are the sole responsibility of the authors. Questions or messages regarding errors should be addressed to the author.

Supplementary Data
