# Supplementary material for: The Diversity of Meningococcal Carriage Across the African Meningitis Belt and the Impact of Vaccination With a Group A Meningococcal Conjugate Vaccine
Source: J Infect Dis. 2015 Apr 9;212(8):1298–307. doi: 10.1093/infdis/jiv211 (PMC4577048; doi:10.1093/infdis/jiv211)
Supplement: Supplementary Data [file supp_212_8_1298__index.html]

The Diversity of Meningococcal Carriage Across the African Meningitis Belt and the Impact of Vaccination With a Group A Meningococcal Conjugate Vaccine — The Diversity of Meningococcal Carriage Across the African Meningitis Belt and the Impact of Vaccination With a Group A Meningococcal Conjugate Vaccine — Supplementary Data 

# The Diversity of Meningococcal Carriage Across the African Meningitis Belt and the Impact of Vaccination With a Group A Meningococcal Conjugate Vaccine

## Supplementary Data

Supplementary Data

**Files in this Data Supplement:**

- Supplementary Data - Docx file
