# Supplementary material for: The Diversity of Meningococcal Carriage Across the African Meningitis Belt and the Impact of Vaccination With a Group A Meningococcal Conjugate Vaccine
Source: J Infect Dis. 2015 Apr 9;212(8):1298–307. doi: 10.1093/infdis/jiv211 (PMC4577048; doi:10.1093/infdis/jiv211)
Supplement: Supplementary Data [file supp_jiv211_jiv211supp.docx]

**SUPPLEMENTARY MATERIALS**

**Contents**

[**1**. **LABORATORY METHODS** 3](#_Toc393961393)

[Isolation and characterisation of meningococci 3](#_Toc393961394)

[Molecular characterisation of *Neisseria* 5](#_Toc393961395)

[References 7](#_Toc393961396)

[**2**. **SUPPLEMENTARY TABLES** 8](#_Toc393961397)

[**Table S1:** Numbers of laboratory reports in total and numbers excluded from main analyses because they could not be matched to any information on the age of the participant. 9](#_Toc393961398)

[**Table S2**: Distribution of subjects recruited to the carriage studies by age and centre. 10](#_Toc393961399)

[**Table S3:** Carriage prevalence by area and cross-sectional survey. 12](#_Toc393961400)

[**Table S4:** Age and sex distribution of carriers by genogroup (all surveys combined) 14](#_Toc393961401)

[**Table S5:** Factors associated with carriage of meningococcci possessing genes encoding a capsule (caps) and capsule null (cnl) meningococci compared to those not carrying any meningococci, results from multinomial logistic regression. 16](#_Toc393961402)

[**3**. **SUPPLEMENTARY FIGURES** 18](#_Toc393961403)

[**Figure S1.** Geographical position of the study centres. 18](#_Toc393961404)

[**Figure S2**: Age-specific carriage prevalence by country and survey. 19](#_Toc393961405)

# **1. LABORATORY METHODS**

## **1.1 Isolation and characterisation of meningococci**

Pharyngeal swabs were plated directly onto Modified Thayer Martin agar plates in the field and taken to the laboratory within six hours of collection where they were incubated for 24 - 48 hours at 37^0^ C in 5% CO_2_. During a pilot study conducted at each centre ([Basta, Stuart et al. 2013](#_ENREF_1)), one or two colonies from the Thayer Martin plate with a morphology typical of *Neisseria* species were selected for Gram staining and oxidase testing (Beckton Dickinson, Oxford, UK). Oxidase positive, gram negative colonies were then sub-cultured onto one blood agar plate containing 5% defibrinated sheep’s blood and these plates were incubated for 18 -24 hours at 37 ^0^C in 5% CO_2_ (Martin *et al*, 1974). Bacteria from the blood agar plate were serogrouped by slide agglutination with meningococcal serogroup A, W, X and Y antisera (Difco, Becton Dickinson, Oxford, UK). A fine suspension of the test organism was made in 10 µL of phosphate buffered saline (PBS) on a clean glass slide and 10 µL of antiserum were added to the bacterial suspension. The slide was rocked for four minutes before the agglutination pattern was read. A number of problems were encountered with this procedure which required modification to the standard operating procedure for the main study. For the main study, one suspected colony with a typical morphology of a *Neisseria* species was sub-cultured onto two blood agar plates and the plates incubated for 18-24 hours at 37^o^ C. Bacteria from one blood agar plate were tested for oxidase activity and then Gram stained. Oxidase positive gram negative diplococci were then tested for γ-glutamyl-transferase activity (GGT) (Rosco Diagnostica, Taastrup, Denmark) specific for *N. meningitidis* (13),β-galactosidase activity with ortho-nitrophenyl-β-D-galactopyranoside (ONPG) (Rosco Diagnostica ,Taastrup, Denmark) for identification of *N. lactamica* and butyrate esterase activity (Tributyrin ) (Rosco Diagnostica ,Taastrup , Denmark) for distinguishing between *N. meningitidis* and *Moraxella* species (Perez *et al*, 1990).To carry out these tests, a loopful of 18-24 hour culture of the test organism grown on blood agar was suspended in 250 µL of normal saline and the suspension incubated with the appropriate enzyme substrate at 37^0^ C for four hours before observation for the indicative colour change. Isolates with the profile GGT positive, ONPG negative and Tributyrin negative were characterised as presumptive *N. meningitidis* and serogrouped by slide agglutination using four serogrouping antisera for Men A, W, X and Y respectively (Difco, Becton Dickinson, Oxford UK) starting with A and W, followed by X and Y if the first two did not generate agglutination. A fine suspension of the test organism was made directly in each antiserum on a clean glass slide and the slide rocked for one minute. Each antiserum was tested with a positive and negative control bacterium. A sample was assigned to a specific serogroup only if visible agglutination or clumping of the bacteria, with clearing of the background bacterial suspension on the slide, was observed within one minute (World Health Organisation, 2009). Bacterial colonies from the second agar plate were used for DNA preparation by boiling the bacteria from half a plate suspended in 1 mL of PBS for 20 minutes in a boiling water bath. The rest of the bacteria on the same agar plate were prepared for long term storage on beads (Microbank, Pro Lab Diagnostics, Bromborough, UK), following the manufacturers’ instructions, and the sample stored at -80^0^ C.

Molecular characterisation of bacteria obtained during the pilot study undertaken at both CERMES, Niamey, Niger and at the University of Oxford showed inaccuracies in both speciation and serogrouping and in preparation of an adequate DNA sample. The first problem was addressed by the introduction of biochemical tests prior to serogrouping/genogrouping, as outlined above. The problem of an inadequate quantity of DNA was resolved by preparing DNA from half a plateful of bacteria grown on a second blood agar plate.

## **1.2 Molecular characterisation of *Neisseria***

DNA suspensions of oxidase positive, Gram negative diplococci, identified as described above, were separated into four aliquots. One aliquot was sent to the University of Oxford for molecular analysis, the second used on site for PCR identification of *N. meningitidis* and the remaining two aliquots archived.

Following a workshop at CERMES, Niamey on PCR detection and genogrouping of meningococci and regular follow-up visits, six of the seven MenAfriCar centres are now able to conduct a *por A* *PCR* test to confirm the identity of isolates of *N. meningitidis* identified by routine microbiology. If the *por A* PCR test is positive, an A, X, W multiplex genogrouping PCR is performed. If this first multiplex PCR is negative, a singleplex Y genogrouping PCR is performed. Ready-to-load master mix containing hotstart Taq polymerase (Solis BioDyne, Tartu, Estonia) was chosen as the PCR mixture to render the PCR technique as simple as possible and because this enzyme-containing master mix can be transported at room temperature. The 7.5 mM MgCl_2_ master mix was changed to 11 mM MgCl_2_ to optimise the technique. Prior to the introduction of PCR testing as a routine procedure, each site underwent cross-validation of the technique, comparing results of approximately 50 samples with results obtained at the University of Oxford.

On arrival in Oxford, heat-killed bacterial DNA suspensions were archived and entered on a BIGSdb database (Maiden & Jolley, 2010; Maiden *et al* 2013) for subsequent hierarchical analysis using a custom built high-throughput Sanger sequencing pipeline. All sequence data were submitted directly into this database and the results interpreted automatically by querying with reference sequences. The first stage of characterisation of *Neisseria* species is done with an assay based on ribosomal Multi Locus Sequence Typing (rMLST) (Jolley *et al* 2012) which indexes variation in a 413bp fragment of one of the 53 ribosomal protein genes encoding ribosomal protein subunit L6 (*rplF*) which is able alone to reproduce the unambiguous discrimination of members of the *Neisseria* species obtained with rMLST ([Bennett *et al.* 2014](#_ENREF_2)). The *rplF* gene fragment is amplified and sequenced. A null result (no sequence) is consistent with the specimen not containing a member of the genus *Neisseria*, while sequences obtained from positive specimens were characterized, based on the alleles phylogeny, as the appropriate *Neisseria* species. Isolates that are *rplF* negative are characterised by sequencing a fragment of the 16S (small subunit) rRNA gene to confirm genus or lack of bacterial DNA suitable for PCR amplification. All samples are then tested with an assay which sequences the capsule null (*cnl*) region of the meningococcal chromosome (Claus *et al* 2012). This region is present in meningococci genetically characterised without a capsule and in all *Neisseria* species other than meningococci. Sequencing of the two variable regions, VR1 and VR2, of the outer membrane protein encoding gene *porA* was also done to confirm the identification and further characterise the *N. meningitidis* isolates. All samples were tested by real-time PCR amplification of the *cap* locus (NmA *sacB*, NmW *synG*, NmX *xcbB*, NmB *synD*, NmC *synE*, and NmY *synF*) which is present in all meningococci with a capsule synthesis region (*cps*) region using two multiplex Real Time PCR assays)([Wang *et al.* 2012](#_ENREF_7)); the first one identified genogroups A, W and X and the second one serogroup B, C and Y.

## **1.3 References**

Basta NE, Stuart JM, Nascimento MC, Manigart O, Trotter C et al (2013). Methods for identifying Neisseria meningitidis carriers: a multi-center study in the African meningitis belt. PLoS One 8(10): e78336.

Bennett JS, Watkins ER, Jolley KA, Harrison OB, Maiden MC. (2014). Identifying Neisseria Species by Use of the 50S Ribosomal Protein L6 (rplF) Gene. J Clin Microbiol 52(5): 1375-1381.

Claus H, Jördens MS, Kriz P, Musilek M, Jarva H, et al. (2012). Capsule null locus meningococci: typing of antigens used in an investigational multicomponent meningococcus serogroup B vaccine. Vaccine 30(2): 155-160.

Jolley KA, Bliss CM, Bennett JS, Bratcher HB, Brehony C, et al. (2012). Ribosomal multilocus sequence typing: universal characterization of bacteria from domain to strain. Microbiology 158(Pt 4): 1005-1015.

Jolley KA, Maiden MC (2010). BIGSdb: Scalable analysis of bacterial genome variation at the population level. BMC Bioinformatics 11: 595.

Maiden MC, van Rensburg MJ, Bray JE, Earle SG, Ford SA, et al. (2013). MLST revisited: the gene-by-gene approach to bacterial genomics. Nat Rev Microbiol 11(10): 728-736.

Martin JE, Armstrong JH, Smith PB (1974) New system for cultivation of Neisseria gonorrhoeae. Applied Microbiology 27, 802–805.

Perez JL, Pulido A, Pantozzi F, Martin R (1990) Butyrate esterase (4-methylumberllifery butyrate) spot test, a simple method for immediate identification of *Moraxella* *(Branhamella) catarrhalis*. Journal of Clinical Microbiology 28, 2347–2348.

Wang X, Theodore MJ, Mair R, Trujillo-Lopez E, du Plessis M, et al. (2012) Clinical validation of multiplex real-time PCR assays for detection of bacterial meningitis pathogens. J Clin Microbiol 50(3): 702-708.

World Health Organization (2009). Laboratory methods for the diagnosis of meningitis caused by *Neisseria meningitidis*, *Streptococcus pneumoniae*, and *Haemophilus influenzae*. 2nd edn, WHO/IVB.11.09, Geneva.

#

#

### **SUPPLEMENTARY TABLES**

### **Table S1:** Numbers of laboratory reports in total and numbers excluded from main analyses because they could not be matched to any information on the age of the participant.

Note that only 18 confirmed meningococci were excluded.

| **Country** | **Survey 1** | | **Survey 2** | | **Survey 3** | |
| --- | --- | --- | --- | --- | --- | --- |
|  | Total lab reports | Exclusions (carriers) | Total lab reports | Exclusions (carriers) | Total lab reports | Exclusions (carriers) |
| Chad | 2002 | 16 (5) | 5339 | 32 (2) | 6185 | 82 (0) |
| Ethiopia | 2005 | 121 (0) | 2046 | 21 (0) | 2066 | 5(0) |
| Ghana | 1194 | 35 (0) | 2039 | 8 (0) | 2020 | 1 (1) |
| Mali | 4987 | 143 (1) | 1995 | 1 (0) | 2002 | 3 (0) |
| Niger | 4282 | 47 (4) | 1964 | 1 (0) | 2019 | 4 (0) |
| Nigeria | 1844 | 324 (1) | 1004 | 68 (0) |  | - |
| Senegal | 1456 | 1456 (4) | 1687 | 7 (0) | 1323 | 8 (0) |
| Total | 17770 | 728 (15) | 16077 | 138 (2) | 15605 | 103 (1) |

### **Table S2**: Distribution of subjects recruited to the carriage studies by age and centre.

| **Country** | **Age group** | **Survey1**  Rainy season,  July to December 2010 | **Survey 2**  Rainy season,  July to November 2011 | **Survey 3**  Dry season,  February to July 2012 |
| --- | --- | --- | --- | --- |
| Chad | 0-4 years (<1 year) | 474 (107) | 1393 (315) | 550 (127) |
|  | 5-14 years | 419 | 1262 | 2747 |
|  | 15-29 years | 511 | 1280 | 2236 |
|  | 30 + years | 582 | 1372 | 570 |
|  | Total | 1986 | 5307 | 6103 |
| Ethiopia | 0-4 years (<1 year) | 494 (110) | 505 (108) | 527 (106) |
|  | 5-14 years | 471 | 489 | 517 |
|  | 15-29 years | 455 | 511 | 503 |
|  | 30 + years | 464 | 520 | 514 |
|  | Total | 1884 | 2025 | 2061 |
| Ghana | 0-4 years (<1 year) | 247 (46) | 399 (41) | 357 (48) |
|  | 5-14 years | 252 | 506 | 502 |
|  | 15-29 years | 283 | 493 | 488 |
|  | 30 + years | 377 | 633 | 672 |
|  | Total | 1159 | 2031 | 2019 |
| Mali | 0-4 years (<1 year) | 1211 (233) | 499 (98) | 497 (99) |
|  | 5-14 years | 1204 | 499 | 503 |
|  | 15-29 years | 1159 | 499 | 498 |
|  | 30 + years | 1270 | 497 | 501 |
|  | Total | 4844 | 1994 | 1999 |
| Niger | 0-4 years (<1 year) | 1000 (183) | 474 (91) | 521 (112) |
|  | 5-14 years | 1012 | 487 | 506 |
|  | 15-29 years | 1059 | 484 | 489 |
|  | 30 + years | 1164 | 518 | 499 |
|  | Total | 4235 | 1963 | 2015 |
| Nigeria | 0-4 years (<1 year) | 390 (66) | 283 (59) | 0 |
|  | 5-14 years | 343 | 215 | 0 |
|  | 15-29 years | 352 | 212 | 0 |
|  | 30 + years | 435 | 226 | 0 |
|  | Total | 1520 | 936 | 0 |
| Senegal | 0-4 years (<1 year) | 404 (84) | 447 (91) | 366 (75) |
|  | 5-14 years | 370 | 450 | 367 |
|  | 15-29 years | 286 | 345 | 282 |
|  | 30 + years | 354 | 438 | 300 |
|  | Total | 1414 | 1680 | 1315 |

### **Table S3:** Carriage prevalence by area and cross-sectional survey (XS1 – XS-3).

|  |  | **Rainy season 2010 (XS1)** | | **Rainy season 2011 (XS2)** | | **Dry season 2012 (XS3)** | | **Overall** | |
| --- | --- | --- | --- | --- | --- | --- | --- | --- | --- |
|  |  | *N sampled* | *Carriage prevalence (%)* | *N sampled* | *Carriage prevalence*  *(%)* | *N sampled* | *Carriage prevalence*  *(%)* | *N sampled* | *Carriage prevalence*  *(%)* |
| Chad | urban | 998 | 0.8 | 1046 | 1.2 | 1108 | 0.7 | 3152 | 0.9 |
|  | rural | 988 | 0.6 | 4261 | 1.3 | 4995 | 0.8 | 10244 | 1.0 |
| Ethiopia | urban | 940 | 5.6 | 1011 | 5.2 | 1034 | 4.3 | 2985 | 5.0 |
|  | rural | 944 | 7.1 | 1014 | 6.8 | 1027 | 10.0 | 2985 | 8.0 |
| Ghana | urban | 557 | 0.2 | 1030 | 0.6 | 1007 | 6.8 | 2594 | 2.9 |
|  | rural | 602 | 0.7 | 1001 | 6.4 | 1012 | 4.9 | 2615 | 4.5 |
| Mali | urban | 2405 | 0.7 | 997 | 1.3 | 999 | 1.4 | 4401 | 1.0 |
|  | rural | 2439 | 0.4 | 997 | 0.6 | 1000 | 2.0 | 4436 | 0.8 |
| Niger | urban | 2433 | 8.0 | 965 | 6.3 | 1002 | 2.0 | 4400 | 6.3 |
|  | rural | 1802 | 9.8 | 998 | 6.3 | 1013 | 2.0 | 3813 | 6.8 |
| Nigeria | urban | 781 | 0.4 | 0 | - | 0 | - | 781 | 0.4 |
|  | rural | 739 | 0.0 | 936 | 0.0 | 0 | - | 1675 | 0.0 |
| Senegal | urban | 706 | 2.0 | 771 | 1.4 | 453 | 11.5 | 1930 | 4.0 |
|  | rural | 708 | 4.4 | 909 | 4.5 | 862 | 24.1 | 2479 | 11.3 |
| TOTAL | urban | 8820 | 3.3 | 5820 | 2.7 | 5603 | 3.7 | 20243 | 3.2 |
|  | rural | 8222 | 3.6 | 10116 | 2.9 | 9909 | 4.5 | 28247 | 3.7 |
|  | TOTAL | 17042 | 3.4 | 15936 | 2.9 | 15512 | 4.2 | 48490 | 3.5 |

### **Table S4:** Age and sex distribution of carriers by genogroup (all surveys combined).

|  | **GROUP A^[[1]](#footnote-1)^** |  |  |
| --- | --- | --- | --- |
| **Age group (years)** | **Female** | **Male** | **Overall** |
| 0-4 | 3 | 2 | 6 |
| 5-14 | 8 | 12 | 21 |
| 15-29 | 4 | 5 | 10 |
| 30+ | 5 | 4 | 9 |
| Total | 20 | 23 | 46 |
|  | **GROUP C** |  |  |
| **Age group (years)** | **Female** | **Male** | **Overall** |
| 0-4 | 1 | 2 | 3 |
| 5-14 | 4 | 3 | 7 |
| 15-29 | 5 | 6 | 11 |
| 30+ | 5 | 4 | 9 |
| Total | 15 | 15 | 30 |
|  | **GROUP W** |  |  |
| **Age group (years)** | **Female** | **Male** | **Overall** |
| 0-4 | 52 | 59 | 111 |
| 5-14 | 103 | 154 | 257 |
| 15-29 | 71 | 77 | 148 |
| 30+ | 78 | 29 | 107 |
| Total | 304 | 319 | 623 |
|  | **GROUP X^[[2]](#footnote-2)^** |  |  |
| **Age group (years)** | **Female** | **Male** | **Overall** |
| 0-4 | 1 | 0 | 1 |
| 5-14 | 5 | 6 | 12 |
| 15-29 | 3 | 3 | 6 |
| 30+ | 2 | 4 | 6 |
| Total | 11 | 13 | 25 |
|  | **GROUP Y** |  |  |
| **Age group (years)** | **Female** | **Male** | **Overall** |
| 0-4 | 0 | 3 | 3 |
| 5-14 | 14 | 9 | 23 |
| 15-29 | 9 | 14 | 23 |
| 30+ | 6 | 13 | 19 |
| Total | 29 | 39 | 68 |
|  | **Capsule null^[[3]](#footnote-3)^** |  |  |
| **Age group (years)** | **Female** | **Male** | **Overall** |
| 0-4 | 58 | 63 | 121 |
| 5-14 | 136 | 159 | 295 |
| 15-29 | 129 | 85 | 216 |
| 30+ | 85 | 61 | 146 |
| Total | 408 | 368 | 778 |

### **Table S5:** Factors associated with carriage of meningococci possessing genes encoding a capsule (caps) and capsule null (cnl) meningococci compared to those not carrying any meningococci, results from multinomial logistic regression.

|  | | **Number of …** | | **Adjusted OR (95% CI)** | |
| --- | --- | --- | --- | --- | --- |
| **Factor** | | **Participants** | **Carriers**  **caps/ cnl** | **Caps** | **Cnl** |
| Age | <1 year | 2199 | 26/14 | 0.18 (0.32, 0.72) | 0.28 (0.16, 0.47) |
|  | 1-4 years | 8839 | 121/106 | 0.61 (0.49, 0.76) | 0.58 (0.46, 0.73) |
|  | 5-14 years | 13121 | 354/292 | 1.49 (1.26, 1.76) | 1.37 (1.14, 1.64) |
|  | 15-29 years | 12424 | 230/211 | 1.0 | 1.0 |
|  | 30+ years | 11907 | 166/141 | 0.60 (0.49, 0.74) | 0.60 (0.48, 0.74) |
| Sex | Female | 27994 | 441/408 | 1.0 | 1.0 |
|  | Male | 20361 | 464/368 | 1.17 (1.10, 1.25) | 1.19 (1.08, 1.31) |
| Season | Rainy (Survey 1,2) | 32978 | 463/576 | 1.0 | 1.0 |
|  | Dry (Survey 3) | 15512 | 446/202 | 2.25 (1.92, 2.65) | 0.97 (0.78, 1.20) |
| Country | Chad | 13396 | 81/50 | 1.0 | 1.0 |
|  | Ethiopia | 5970 | 85/304 | 2.18 (1.45, 3.27) | 15.6 (10.98, 22.29) |
|  | Ghana | 5209 | 179/14 | 6.30 (4.39, 9.02) | 1.02 (0.56, 1.86) |
|  | Mali | 8837 | 61/18 | 1.86 (1.23, 2.79) | 0.82 (0.47, 1.43) |
|  | Niger | 8213 | 201/334 | 6.67 (4.77, 9.34) | 15.15 (10.89, 21.08) |
|  | Nigeria | 2456 | 3/0 | 0.20 (0.05, 0.84) | - |
|  | Senegal | 4409 | 299/58 | 12.13 (8.40, 17.54) | 4.82 (3.17, 7.33) |
| Area | Urban | 20243 | 361/291 | 1.0 | 1.0 |
|  | Rural | 28247 | 548/487 | 1.30 (1.11, 1.51) | 1.62 (1.34, 1.96) |
| Crowded | <2 people per room | 16903 | 294/166 | 1.0 | 1.0 |
|  | ≥ 2 people per room | 31520 | 603/598 | 1.21 (1.03, 1.44) | 1.30 (1.06, 1.58) |
| Kitchen location | Open air | 18935 | 234/278 | 1.0 | 1.0 |
|  | Inside house | 13322 | 485/339 | 1.56 (1.19, 2.05) | 1.10 (0.85, 1.43) |
|  | Separate hut | 15571 | 171/133 | 1.10 (0.83, 1.45) | 0.85 (0.61, 1.19) |
|  | Missing | 595 | 7/14 | 0.75 (0.30, 1.86) | 1.50 (0.74, 3.03) |
| Vaccinated recently with meningitis vaccine | No | 33599 | 636/594 | 1.0 | 1.0 |
|  | Yes, <1 year ago | 9131 | 110/117 | 0.61 (0.48, 0.77) | 0.88 (0.69, 1.11) |
|  | Yes, 1-3 years ago | 4589 | 125/46 | 0.60 (0.48, 0.76) | 0.44 (0.30, 0.65) |
|  | Don’t know/ missing | 1076 | 25/7 | 0.87 (0.56, 1.34) | 0.51 (0.24, 1.09) |

# **3. SUPPLEMENTARY FIGURES**

### **Figure S1.** Geographical position of the study centres.

### **Figure S2**: Age-specific carriage prevalence by country and survey.

1. First cross-sectional survey


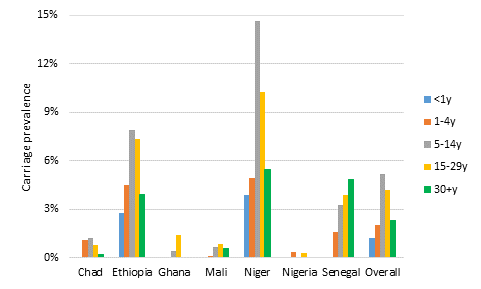


1. *Second cross-sectional survey*

*
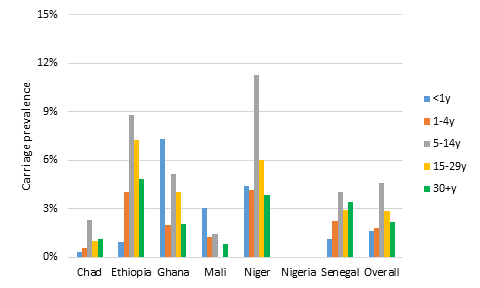
*

1. *Third cross sectional survey (note that the y-axis is higher compared to panels a and b)*

*
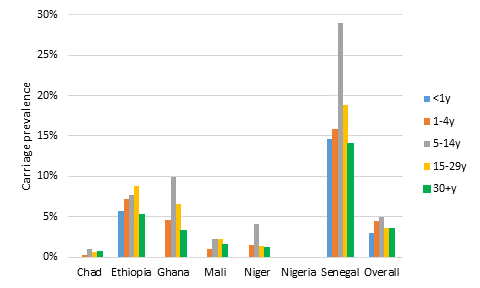
*

1. 3 group A with missing sex [↑](#footnote-ref-1)
2. 1 group X with missing sex [↑](#footnote-ref-2)
3. 2 cnl with missing sex [↑](#footnote-ref-3)
